# Skillful Cycling Training Induces Cortical Plasticity in the Lower Extremity Motor Cortex Area in Healthy Persons

**DOI:** 10.3389/fnins.2019.00927

**Published:** 2019-09-03

**Authors:** Tsuyoshi Tatemoto, Satoshi Tanaka, Kazuhei Maeda, Shigeo Tanabe, Kunitsugu Kondo, Tomofumi Yamaguchi

**Affiliations:** ^1^Graduate School of Health Sciences, Fujita Health University, Aichi, Japan; ^2^Laboratory of Psychology, Hamamatsu University School of Medicine, Shizuoka, Japan; ^3^Department of Clinical Technology, Hokkaido Institutional Society Otaru Hospital, Hokkaido, Japan; ^4^Faculty of Rehabilitation, School of Health Sciences, Fujita Health University, Aichi, Japan; ^5^Tokyo Bay Rehabilitation Hospital, Chiba, Japan; ^6^Department of Physical Therapy, Yamagata Prefectural University of Health Sciences, Yamagata, Japan

**Keywords:** short-interval intracortical inhibition, lower extremity, motor learning, cortical plasticity, cycling, rehabilitation

## Abstract

Cycling exercise is commonly used in rehabilitation to improve lower extremity (LE) motor function and gait performance after stroke. Motor learning is important for regaining motor skills, suggesting that training of motor skills influences cortical plasticity. However, the effects of motor skill learning in dynamic alternating movements of both legs on cortical plasticity remain unclear. Here, we examined the effects of skillful cycling training on cortical plasticity of the LE motor area in healthy adults. Eleven healthy volunteers participated in the following three sessions on different days: skillful cycling training, constant-speed cycling training, and rest condition. Skillful cycling training required the navigation of a marker up and down curves by controlling the rotation speed of the pedals. Participants were instructed to fit the marker to the target curves as accurately as possible. Amplitudes of motor evoked potentials (MEPs) and short-interval intracortical inhibition (SICI) evoked using transcranial magnetic stimulation (TMS) were assessed at baseline, after every 10 min of the task (a total of 30 min), and 30 min after the third and final trial. A decrease in tracking errors was representative of the formation of motor learning following skillful cycling training. Compared to baseline, SICI was significantly decreased after skillful cycling training in the tibialis anterior (TA) muscle. The task-induced alterations of SICI were more prominent and lasted longer with skillful cycling training than with the other conditions. The changes in SICI were negatively correlated with a change in tracking error ratio at 20 min the task. MEP amplitudes were not significantly altered with any condition. In conclusion, skillful cycling training induced long-lasting plastic changes of intracortical inhibition, which corresponded to the learning process in the LE motor cortex. These findings suggest that skillful cycling training would be an effective LE rehabilitation method after stroke.

## Introduction

Motor impairments following stroke remain one of the leading causes of long-term disability in daily life (Miller et al., 2010; Lee and Cho, 2017). There is substantial evidence that rehabilitative training such as constraint-induced movement therapy promotes cortical plasticity (Mark et al., 2006), and that plastic changes in the motor cortex, as measured by transcranial magnetic stimulation (TMS) and functional neuroimaging, are related to functional recovery of the upper extremity in stroke patients (Choo et al., 2015; Beaulieu and Milot, 2018). Cortical plasticity following rehabilitative training plays an important role in recovery of motor function (Nudo, 1997).

Cycling exercise has been proposed as an effective approach to improve lower extremity (LE) motor function and gait performance in patients with stroke (Brown and Kautz, 1998; Fujiwara et al., 2003; Brown et al., 2005; Ferrante et al., 2011). Fujiwara et al. (2003) reported that phasic muscle activity is induced in the affected LE during cycling training. They also found that muscle activity in the quadriceps femoris and tibialis anterior (TA) was significantly increased after cycling training in chronic stroke patients. Furthermore, Ferrante et al. (2011) have reported that a 2 week regimen of cycling training improved gait speed and asymmetry in patients with chronic stroke. Neuroimaging studies have shown that motor related cortical areas are activated during cycling exercise (Christensen et al., 2000; Pyndt and Nielsen, 2003; Mehta et al., 2009; Promjunyakul et al., 2015). Neurophysiological studies have reported the changes of H-reflex, reciprocal inhibition and short-interval intracortical inhibition (SICI) after cycling exercise in healthy persons (Mazzocchio et al., 2006; Yamaguchi et al., 2012, 2013) and patients with stroke (Tanuma et al., 2017). These studies suggest that cycling exercise may induce neural plasticity which contributes to functional recovery in the LE. However, the relationship between neural mechanisms that enhance cortical plasticity of the LE and motor learning of bipedal performance is unclear.

Motor learning is important for regaining motor skills including gait, and motor skill training may influence cortical plasticity after brain injury (Nudo, 1997). Pharmacological and neurophysiological studies have suggested the involvement of inhibitory interneuronal circuits reflected by altered intracortical γ-aminobutyric acid (GABA)-ergic transmission (Matsumura et al., 1991, 1992; Bütefisch et al., 2000; Lech et al., 2001). In fact, pretreatment with a GABA receptor agonist resulted in a significant reduction in the effects of motor training (Tegenthoff et al., 1999; Bütefisch et al., 2000; Ziemann et al., 2001; Floyer-Lea et al., 2006), showing the functional relevance of GABA-based systems in motor training. GABAergic inhibitory systems can be examined with the use of paired-pulse TMS (Kujirai et al., 1993; Ziemann et al., 1996). Indeed, Perez et al. (2004) reported that skillful motor training with tracking tasks controlled by plantar dorsiflexion of unilateral ankle joints induced a reduction in SICI in motor learning assessed using the paired-pulse TMS method.

Motor learning of coordinated alternating movements of both legs, such as in cycling, is important to efficiently reacquire gait performance following stroke. A functional MRI study by Marchal-Crespo et al. (2017) revealed that gait-like motor learning depends on the interplay between subcortical, cerebellar, and fronto-parietal brain regions including the primary motor cortex during robotic bilateral training. However, no studies to date have investigated alterations in intracortical inhibition with the learning of dynamic bilateral alternating exercises. We hypothesized that progress in motor learning would induce on-going cortical plastic changes with the implementation of skillful training to an exercise that involved alternating movements of both legs (Mazzocchio et al., 2006). In this study, we examined the effects of a cycling motor task which incorporates skillful tracking via the adjustment of rotational speed on cortical plasticity using paired-pulse TMS.

## Materials and Methods

### Participants

Eleven healthy volunteers participated in this study (eight males; mean age ± standard deviation, 25.4 ± 2.5). Sample size was determined based on previous studies investigating the effects of cycling exercise or ankle exercise on intracortical inhibition (Perez et al., 2004; Yamaguchi et al., 2012). Exclusion criteria were a history of neurological diseases, orthopedic problems in the LE, severe cardiac disorders or receiving any medications which affect the central nervous system. All participants provided written informed consent prior to participation in the study. The experimental procedures were approved by the Ethics Committee of the Tokyo Bay Rehabilitation Hospital and conformed to the requirements of the Declaration of Helsinki.

### Experimental Paradigm

**FIGURE 1:**
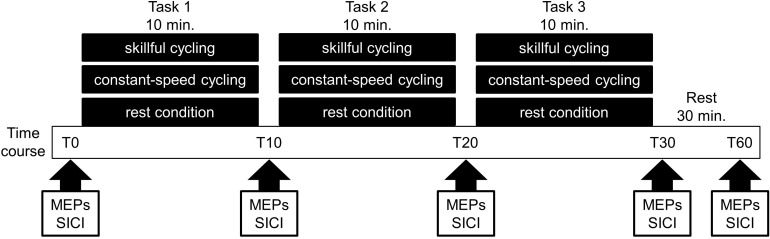
Experimental protocol. Eleven volunteers participated in the following three sessions on different days: (1) skillful cycling training, (2) constant-speed cycling training, and (3) rest condition. During each condition, the motor evoked potentials (MEPs) and short-interval intracortical inhibition (SICI) were measured at baseline (T0), 10 min (T10), 20 min (T20), 30 min (T30), and 60 min (T60) after the start of the experiment.

The present study employed a randomized crossover design. All participants performed the following sessions on different days: (1) skillful cycling training, (2) constant-speed cycling training, and (3) rest condition (see Figure 1). The task order was counterbalanced among participants. To prevent carry-over effects from previous interventions, washout intervals of 1 week or more were implemented between sessions in all participants.

### Tasks

Participants were comfortably seated on a servo-dynamically controlled recumbent ergometer (StrengthErgo240, Mitsubishi Electric Co., Japan). Their feet were firmly strapped to the pedals and a seat belt and adjustable backrest with a tilt angle of 80° was used to stabilize their trunk. The ergometer used was able to achieve a highly precise load control (coefficient of variation, 5%) over a wide range of cycling resistances (0–240 Nm). The ergometer seat and crank heights were set at 51 and 17 cm, respectively. The distance from the seat edge to the crank axis and the height of the pedal axis were adjusted so that the knee extension angle was −10° during maximal extension. An isotonic mode was utilized with load sets at 5 Nm (Fujiwara et al., 2003). The load was determined according to previous studies at a setting which could be achieved even by stroke patients with leg motor paralysis (Fujiwara et al., 2003; Tanuma et al., 2017).

#### Skillful Cycling Training

**FIGURE 2:**
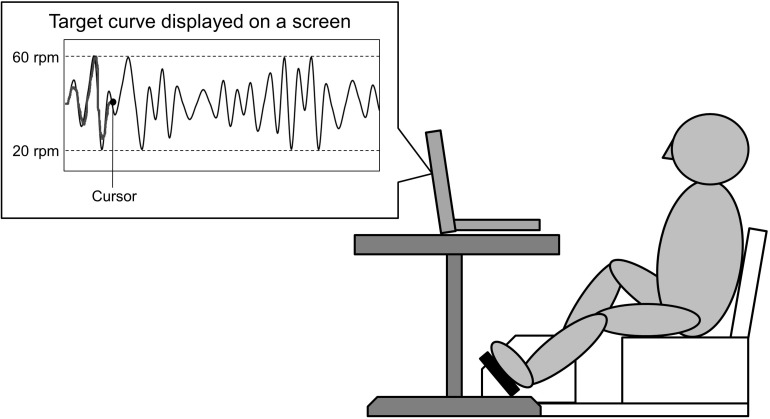
Experimental setting. A schematic diagram of the task is shown. The participants controlled the movement of a cursor on the screen by changing the pedaling speed. Pedaling movements made the cursor move upward. The participants were required to adjust the speed at which the pedals revolved to match the cursor to the target curve displayed on a screen.

Participants performed skillful cycling training, whereby they controlled the movement of a cursor on a computer screen by adjusting the pedaling speed in order to track a marker to target curves (see Figure 2). Pedaling movements caused the cursor to move upward. Participants were instructed to match the cursor (a dot) to the target curves on the screen as accurately as possible by changing the pedaling speed. Participants received real time feedback on the screen which represented the difference between the cursor and the target curves. The displayed waveform was set to a minimum value of 20 revolutions per minutes (rpm), maximum value of 60 rpm, and average pedaling speed of 40 rpm. During skillful cycling, participants were instructed to perform 10 min of cycling for each of three trials (termed Task 1, Task 2, and Task 3). Motor performance was evaluated based on the area of error between the target-tracking waveform and the position of the dot. The area of error was presented as arbitrary units. A custom-written computer program (LabVIEW software, ver. 7.1; National Instruments Corp., Austin, TX, United States) was used to design the tracking task and connect the ergometer to the computer.

#### Constant-Speed Cycling Training

The ergometer settings were identical to during skillful cycling training. To control the amount of exercise, a trial required a constant pedaling speed of 40 rpm for 10 min. Using a similar program to the one used during skillful cycling training, participants maintained the appropriate number of rotations while observing a tracking line set at 40 rpm.

#### Rest Condition

As a control, a 10-min rest condition was carried out whereby participants sat on the ergometer in the same manner as during other conditions, but did not engage in cycling.

### Electromyogram (EMG) Recording

Prior to electrode attachment, the area of skin over the recording area of the target muscle was cleansed with alcohol. Throughout the experiments, skin resistance was kept below 5 kΩ. Surface electrodes were placed on the skin overlying the left TA in a bipolar montage (inter-electrode distance of 20 mm). A Neuropack^TR^ electromyography machine (Nihon Kohden Co., Tokyo, Japan) was used to record and analyze the EMG data. A band pass filter was applied between 30 Hz and 2 kHz. Signals were recorded at a sampling rate of 5 kHz and stored on the computer for subsequent analysis using LabVIEW software.

### Transcranial Magnetic Stimulation

Participants seated on an ergometer with a backrest in a relaxed position with 80° hip flexion, 80° knee flexion, 10° ankle plantar flexion, and their feet on the floor. TMS was performed using a magnetic stimulator (Magstim200, Magstim, Dyfed, United Kingdom) capable of delivering a magnetic field of 2.2 T with 100 μs pulse duration through a double cone coil. Each cone had a diameter of 110 mm. The stimulating coil was located 0–2 cm posterior to the vertex and was placed over the site that was optimal for eliciting responses in the left TA and oriented so that the current in the brain flowed in a posterior to anterior direction through this site (Madhavan et al., 2010; Kesar et al., 2018; Škarabot et al., 2019). Since the direction of current flow can affect the motor evoked potential (MEP) responses (Terao et al., 1994, 2000) and the distance from the coil to the cortex affects the MEP amplitude (Stokes et al., 2005), we positioned the double-cone coil to closely conform with the scalp.

The rationale for choosing TA as the target muscle was mainly for the technical reasons that TMS over M1 can induce reliable MEPs from TA (Petersen et al., 2003; Groppa et al., 2012; Kesar et al., 2018). The threshold was determined the TA was at rest, and during voluntary contractions. The threshold was defined as the minimum stimulus intensity that evoked responses of approximately 100 μV with a similar shape and latency in 5 out of 10 successive stimuli. Each participant was requested to relax during measurement of the resting motor threshold (rMT) during which EMG silence was monitored. To determine the active motor threshold (aMT), participants held a muscle contraction at an intensity of 5–10% of their maximum with the help of visual feedback from the EMG.

The intensity of single-pulse TMS was set at 120% of the rMT to measure MEPs as an indicator of corticospinal excitability. A total of 10 MEPs were recorded in the rest condition. Peak-to-peak amplitudes were averaged for each time point. Ten measurements of the peak-to-peak MEP amplitude were averaged, and the mean value and standard error among subjects were calculated.

In the present study, we sought to evaluate cortical plasticity by measuring changes in SICI after the cycling training (Perez et al., 2004; Yamaguchi et al., 2012; Sidhu et al., 2013). In order to induce SICI, we applied sub-threshold conditioning paired-pulse stimulation (Kujirai et al., 1993). Two magnetic stimuli were supplied via the same stimulating coil to the right primary motor cortex. We used 80% of the aMT for the conditioning stimulus and 120% of the rMT for the test stimulus. Throughout the experiment, the intensity of test pulse was adjusted to induce MEPs of equivalent amplitude to prior to the intervention in the relaxed TA. The inter-stimulus interval in the current experiment was set at 2 ms, and 20 frames each were recorded of the paired-pulse and single stimulation conditions for each trial (Yamaguchi et al., 2012). Stimuli were applied every 5 s in pseudorandom order by a laboratory computer programed by LabVIEW software. Amplitude of SICI during the paired-pulse protocol was calculated as the average conditioned MEP amplitude expressed as a percentage of the average unconditioned MEP amplitude (Massé-Alarie et al., 2016). SICI values of 1 therefore represents no inhibition. Evaluation of corticospinal excitability and SICI was performed before cycling (Time 0, T0), immediately after each trial (T10, T20, T30), and 30 min after the third trial (T60).

### Statistical Analyses

We compared the total number of pedal rotations during skillful and constant-speed cycling using two-factor repeated-measures analysis of variance (ANOVA) to analyze the effects of “trial” (Task 1, Task 2, Task 3) and “condition” (skillful cycling training, constant-speed cycling training). Additionally, to compare the degree of arousal between conditions, we compared heart rate data recorded after the skillful and constant-speed cycling using a paired *t*-test.

To confirm the occurrence of motor learning following skillful cycling training, a one-factor repeated-measures ANOVA was performed to analyze the change in area of error between the three trials.

To analyze MEP amplitude and SICI, two-factor repeated measures ANOVA was used to analyze the effects of cycling “time” (T0, T10, T20, T30, T60) and “condition” (skillful cycling training, constant-speed cycling training, rest condition) and any interaction. One-way ANOVA was performed to compare MEP amplitude and SICI between each condition using T0 as a baseline. When analyzing SICI, in order to confirm that the test MEP was not different between trials and conditions, we performed two-factor repeated measures ANOVA using the statistical model described above. A paired *t*-test with Bonferroni’s correction for multiple comparisons was used for *post hoc* analysis if a given ANOVA showed a significant interaction. Retrospective power calculations were performed for paired *t*-tests, with an effect size represented by Cohen’s *d*.

To investigate the relationship between plastic changes in SICI and motor learning, we calculated the tracking error ratio and SICI ratio and correlations between them were assessed using Pearson’s correlation analysis, after checking for normal distribution of the data with the Shapiro–Wilk test. The tracking error ratio values were calculated by dividing values of Task 2 and Task 3 by the value of Task 1. The SICI ratio was calculated as the SICI values of T20 and T30 divided by the value of T10 in order to minimize the exercise-induced changes in SICI values at each time point. All statistical analyses were conducted using IBM SPSS statistics 21 for Windows (SPSS Inc., Chicago, IL, United States). Statistical significance was set at a value of *P* < 0.05 for all tests.

## Results

### Total Number of Pedal Revolutions and Analysis of Physical Conditions

The average number of rotations of the pedals during the skillful and constant-speed cycling conditions was 444.9 ± 4.0 and 448.4 ± 4.4, respectively (mean ± standard error). Two-factor repeated measures ANOVA did not reveal a significant interaction (*F*_2__,__20_ = 2.613, *P* = 0.098) nor any significant main effect (trial: *F*_2__,__20_ = 0.421, *P* = 0.662; condition: *F*_1__,__10_ = 0.351, *P* = 0.567). No participants complained of fatigue after cycling for each condition. There were no significant differences in heart rate after training between the skillful and constant-speed cycling conditions [mean heart rate ± standard deviation for skillful cycling = 75.0 ± 8.0; constant = 72.5 ± 6.2, *t*(10) = 1.63, *P* = 0.135]. These results indicate that there was no difference in the amount of exercise or arousal between the two conditions or between trials.

### Performance Test

**FIGURE 3:**
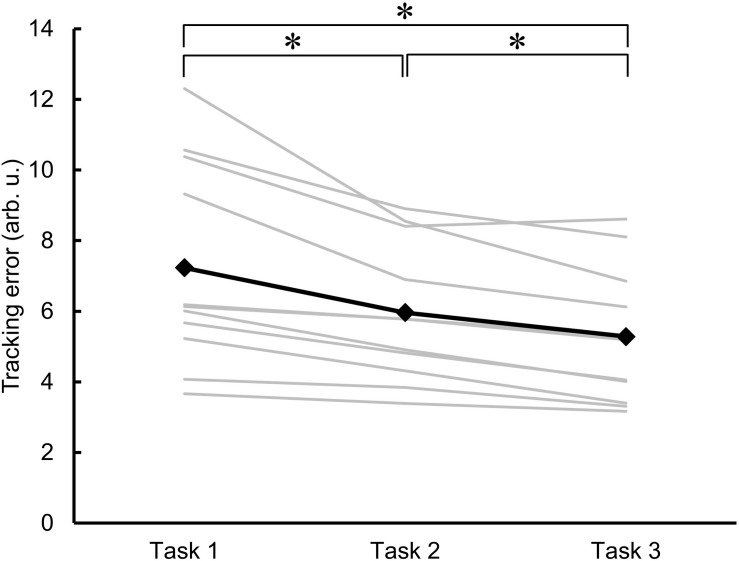
Changes in motor performance. The errors in task performance during the skillful cycling session in Task 1, 2, and 3 are expressed as the area of error in arbitrary units (arb. u.). The gray lines represent individual participants. The black line and markers represent the mean data of all participants. One-factor repeated-measures ANOVA, *N* = 11, ^∗^*P* < 0.05.

Figure 3 shows the individual and mean data for the area of error as an indicator of motor learning across the three trials of skillful cycling training. One-factor repeated-measures ANOVA revealed a significant main effect (*F*_2__,__20_ = 18.829, *P* < 0.001). *Post hoc* test revealed that the area of error for the value of Task 2 and Task 3 was significantly smaller than the value of Task 1 (vs. Task 2, *P* = 0.010; vs. Task 3, *P* = 0.003). Additionally, the area of error of Task 3 was smaller than that of Task 2 (*P* = 0.002). The variance of the individual performance was large at baseline, but gradually decreased with the skillful cycling training (Figure 3).

### MEP Amplitudes

**FIGURE 4:**
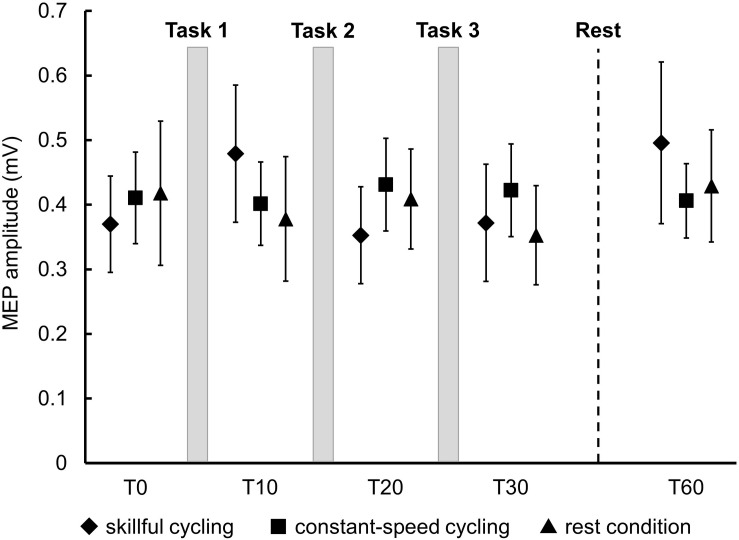
Changes in MEP amplitudes. Each marker represents the mean MEP amplitudes in the tibialis anterior of all participants. Error bars represent standard error.

There was no significant main effect in the baseline of MEP amplitudes between the three conditions (*F*_2__,__20_ = 0.150, *P* = 0.862). Two-factor repeated measures ANOVA did not reveal a significant interaction (*F*_8__,__80_ = 1.383, *P* = 0.217) or any significant main effect (time: *F*_4__,__40_ = 1.723, *P* = 0.164; condition: *F*_2__,__20_ = 0.042, *P* = 0.959) (see Figure 4). These results indicated that a consistent trend for corticospinal excitability was not confirmed for any conditions including skillful cycling training.

### SICI

**FIGURE 5:**
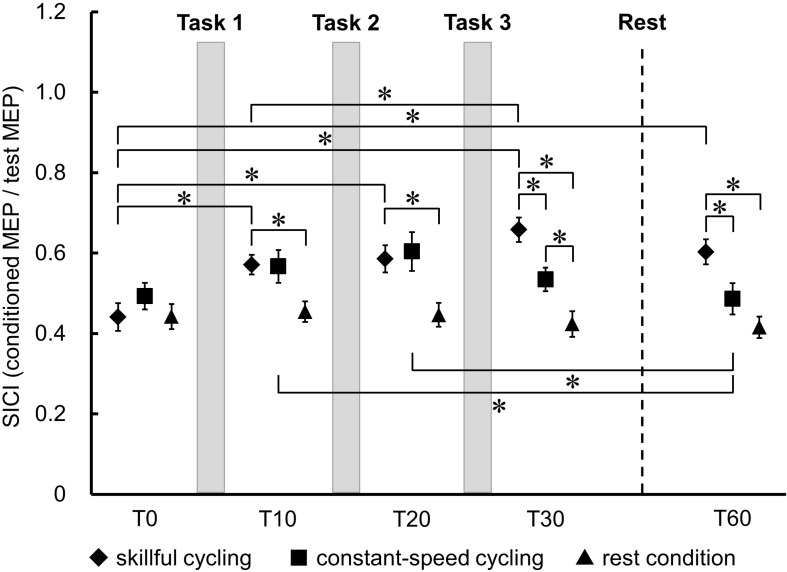
Temporal changes in SICI between each condition. Each marker represents the mean short-interval intracortical inhibition (SICI) in the tibialis anterior. Error bars represent standard error. Two-factor repeated measures ANOVA, *N* = 11, ^∗^*P* < 0.05.

Figure 5 shows the temporal changes and the comparison of SICI in TA between each condition. There was no significant main effect in the baseline of SICI between the three conditions (*F*_2__,__20_ = 1.083, *P* = 0.358). A significant interaction was observed between each the time and condition (*F*_8__,__80_ = 8.793, *P* < 0.001). There were significant main effects of time (*F*_4__,__40_ = 15.005, *P* < 0.001) and condition (*F*_2__,__20_ = 8.318, *P* = 0.002). *Post hoc* testing of the temporal change results revealed that SICI was decreased at all time points relative to T0 in skillful cycling training (vs. T10: Cohen’s *d* = 1.311, power = 0.832; vs. T20: Cohen’s *d* = 1.282, power = 0.816; vs. T30: Cohen’s *d* = 2.002, power = 0.994; vs. T60: Cohen’s *d* = 1.489, power = 0.913). There was a significant difference between T10 and T30 (Cohen’s *d* = 0.942, power = 0.557). In constant-speed cycling training, SICI was significantly decreased at T10 (Cohen’s *d* = 0.609, power = 0.275) and T20 (Cohen’s *d* = 0.807, power = 0.437) compared to T60. Comparisons between conditions revealed that SICI was significantly decreased in skillful cycling training compared to that in the rest condition at T10 or later (T10: Cohen’s *d* = 1.410, power = 0.882; T20: Cohen’s *d* = 1.328, power = 0.842; T30: Cohen’s *d* = 2.257, power = 0.999; T60: Cohen’s *d* = 1.955, power = 0.992). Furthermore, at T30 and T60, SICI for skillful cycling training was significantly decreased compared to that for constant-speed cycling training (T30: Cohen’s *d* = 1.236, power = 0.788; T60: Cohen’s *d* = 1.000, power = 0.607) (see Figure 5). These results suggest that cycling training induced sustained plastic changes in the primary motor cortex, and that these changes were more profound in the skillful cycling than the constant-speed cycling training.

### Correlations Between Tracking Error and SICI

There was a significant negative correlation between the tracking error ratio and the SICI ratio measured after Task 2 (*r* = −0.614, *P* = 0.044). However, there was no correlation after Task 3 (*r* = −0.134, *P* = 0.695).

## Discussion

In the present study, we demonstrated for the first time that skillful cycling training enables motor skill learning for dynamic alternating movements of both legs, which induces long-lasting plastic changes of intracortical inhibition in the LE area of the motor cortex. These findings indicate that skillful cycling training would be more effective than conventional cycling as a neurorehabilitation method.

We adopted a tracking task to adjust the revolution speed of the pedals as a novel skill task for participants. Even when the number of pedal rotations was controlled, there was a significant difference in SICI changes between skillful and constant-speed cycling. Yamaguchi et al. (2012) reported that 7 min of constant-speed cycling reduced SICI in both TA and soleus (SOL) immediately after cycling. Another study reported that 32 min of motor skill training using a tracking task adjusting unilateral ankle joint dorsiflexion or plantar flexion movement induced a reduction in SICI; these effects persisted for 15 min after training (Perez et al., 2004). In our study, 30 min of intermittent skillful cycling induced a reduction in SICI for at least 30 min after training, but these changes were not observed after constant-speed cycling training. These results support the reproducibility of previous results, and suggest that skillful cycling training, which required learning of dynamic alternating movements of both legs, can more effectively induce cortical plastic changes than an equivalent amount of constant-speed cycling. Given the short-term plastic changes in SICI following constant-speed cycling training and lack of changes following passive cycling exercise (Yamaguchi et al., 2012), motor control and skill learning elements may be necessary to induce long-lasting plastic change in SICI in addition to sensorimotor integration. These findings suggest that skillful cycling training may be an effective rehabilitation method for gait disorders. This is supported by previous reports demonstrating that skillful training which require frequent changes in sensory input, led to greater effects than constant training or rest on acquisition of locomotor-related skills (Lam and Pearson, 2002; Mazzocchio et al., 2006).

Alternatively, as GABA is closely involved in control of arousal and sleep (e.g., Saper and Fuller, 2017), it can be argued that the decrease in SICI observed in the present study may reflect non-learning effects such as an increase in arousal after exercise. However, there were no significant differences in heart rate after the training between skillful and constant cycling conditions. This suggests no difference in arousal between the conditions. Therefore, a difference in arousal between the conditions cannot explain the reduction of SICI with the skillful cycling training.

We measured the SICI up to 30 min after the end of pedaling. However, the SICI change in the skillful condition did not return to the baseline at the last measurement of the experiment. Perez et al. (2004) have reported that the reduction of SICI was diminished 15–32 min after skillful leg movement training. Taken together, we speculate that the change in SICI could occur immediately after training but may disappear within a few hours after training.

Several studies have reported that modulation of SICI contributes to motor skill acquisition (Pascual-Leone et al., 1995; Liepert et al., 1998; Classen et al., 1999; Perez et al., 2004). Zimerman et al. (2012) reported that a significant correlation was observed between performance improvement during sequential skillful training and changes in SICI when transcranial direct current stimulation was applied to stroke patients. These phenomena were described as rewiring processes in M1 during acquisition of a novel motor skill, which are most likely based on unmasking of pre-existing connections within the cortex, allowing rapid changes in sensorimotor representations by reducing the activity of existing inhibitory connections (Mengia et al., 1998; Zimerman et al., 2012). The modulation of SICI may also reflect functional input from the cerebellum to M1 during learning (Daskalakis et al., 2004). These findings may support our observation of a significant correlation between the learning acquisition process and changes in SICI. However, the correlation was only present immediately after 20 min of skillful cycling exercise. Coxon et al. (2014) proposed that motor learning may be associated with disinhibition through reduction of SICI with paired-pulse TMS after repetitive pinch force training, particularly in the early acquisition stage (Coxon et al., 2014). The temporal relationship between the learning acquisition process and changes in SICI remains unclear. Thus, the present study provides novel findings on motor learning and cortical plasticity in the LE.

In the present study, no significant increases of MEP amplitude were observed with any cycling conditions. As well as the present study, several previous studies have reported no significant changes in MEP amplitude after LE motor training (Perez et al., 2004; Mazzocchio et al., 2006). Why was no significant increase in MEP amplitude observed? One possibility is that the increase in cortical excitability may be masked by a decrease of excitability at spinal levels when MEP is used as an outcome. Mazzocchio et al. (2006) reported that cycling training induced a decrease in H-reflex amplitude without any significant changes in MEP. Tanuma et al. (2017) reported that the Hmax/Mmax ratio (maximum group I reflex response/maximum direct muscle motor response) was significantly decreased after cycling training. These studies show that the decrease of the spinal excitability occurs after cycling training. Thus, as MEPs are considered to evaluate the total amount of cortical and spinal excitability, the decrease of the spinal excitability may mask the increase of the cortical excitability even if it exists.

While, we did not measure EMG activity during cycling in the present study, previous studies have examined EMG activity in the knee and ankle joints during cycling (Baum and Li, 2003; Fujiwara et al., 2003; da Silva et al., 2016; Roy et al., 2018; Ando et al., 2019). For example, Roy et al. (2018) measured EMG activities from the rectus femoris (RF), biceps femoris, TA, and gastrocnemius muscles during cycling and found that the EMG of the TA (1.5 V) during cycling was similar to that of the RF (1.7 V). What is the role of the TA during cycling? Momeni et al. (2014) have discussed the different roles of the RF and the TA during cycling. They state that the primary function of the RF during cycling is to generate energy in the extension phase, while the energy generated in the limb is transferred to the crank by the TA in the flexion phase (Momeni et al., 2014). Therefore, the change in the cortical plasticity after the skillful cycling training that we observed might be associated with the acquisition of the more sophisticated movement of the TA for these functions.

Cycling has been proposed as an effective approach to improve LE motor function and gait performance in patients with stroke (Brown and Kautz, 1998; Fujiwara et al., 2003; Brown et al., 2005; Ferrante et al., 2011; Promjunyakul et al., 2015; Tanuma et al., 2017). Here, we demonstrated that skillful cycling training could efficiently induce changes in intracortical inhibition in M1. Plastic changes in the cerebral cortex play an important role in regaining motor skills (Zimerman et al., 2012). Therefore, skillful cycling training alone may be useful for stroke patients. Alternatively, several studies reported that cycling training combined with functional electrical stimulation (FES) can improve walking and balancing abilities compared to cycling training without FES in stroke patients (Ambrosini et al., 2011; Bauer et al., 2015; Iyanaga et al., 2019). From these findings, further effects could be expected by applying a method for adjusting afferent sensory input via FES to skillful cycling training.

Several limitations of this study should be noted. First, the sample size of the current study was relatively small, although similar to prior studies targeting LE muscles (Perez et al., 2004; Yamaguchi et al., 2012). Hence, some marginal results, e.g., close to the cutoff for the correlation between the tracking error ratio and SICI ratio (*P* = 0.044), should be interpreted with caution. In the future, we will investigate based on power analysis with enhanced detection power. Second, present results showed no differences in the total number of pedal revolutions and that physical conditions were not different. However, we did not measure EMG activities to investigate the exercise load differences between skillful cycling and constant-speed cycling training, which could have affected the results. Further study is needed to clarify the effects of exercise load on cortical plasticity. Another limitation is that the present study included only healthy adults. The relationship between decreased SICI in spastic patients and improved performance requires further investigation. To verify the effectiveness of this method, studies on stroke patients are required.

## Conclusion

Our study revealed that skillful cycling training which involves a learning task for both legs induced a significant reduction in SICI in the LE motor cortex area compared with conventional cycling. The effects lasted for at least 30 min after training. The current findings provide insight into our understanding of the relationship between cortical plasticity and motor learning in leg performance which could be applied to improve gait function in patients with stroke. In the future, the efficacy of skillful cycling training should be examined in stroke patients as a means to improve gait disorder.

## Data Availability

The datasets generated for this study are available on request to the corresponding author.

## Ethics Statement

Human Subject Research: The studies involving human participants were reviewed and approved by the Ethics Committee of the Tokyo Bay Rehabilitation Hospital. The patients/participants provided their written informed consent to participate in this study.

## Author Contributions

SaT and TY conceived and supervised the study. TT, SaT, and TY designed the experiments and wrote the manuscript. TT, KM, and KK carried out the experiments. ShT constructed the computer program. TT and KM analyzed the data. All authors approved the final version of the submitted manuscript.

## Conflict of Interest Statement

The authors declare that the research was conducted in the absence of any commercial or financial relationships that could be construed as a potential conflict of interest.
